# Pseudoaneurysm after aortopulmonary window repair and bilateral lung transplantation for eisenmenger syndrome: a case report

**DOI:** 10.1186/s13019-023-02305-2

**Published:** 2023-07-01

**Authors:** Toshiaki Nagashima, Masaki Taira, Moyu Hasegawa, Yosuke Kugo, Takuji Watanabe, Daisuke Yoshioka, Kazuo Shimamura, Takayoshi Ueno, Shigeru Miyagawa

**Affiliations:** Department of Cardiovascular Surgery, Osaka University Graduate School of Medicine, 2-2, Yamada-Oka, Suita, 565-0871 Osaka Japan

**Keywords:** Eisenmenger syndrome, Aortopulmonary window, Lung transplantation, Pseudoaneurysm

## Abstract

**Background:**

Aortopulmonary window (APW) is a rare congenital cardiac anomaly characterized by communication between the main pulmonary artery and ascending aorta. There are various surgical techniques, and the short- and long-term results are excellent if the surgical repair is performed early in life. To our knowledge, there have been no reports of pseudoaneurysm after APW repair. Herein, we present a case of a 30-year-old woman with an ascending aortic pseudoaneurysm found at the site of APW repair nine months after the APW repair and bilateral lung transplantation.

**Case presentations:**

A 30-year-old woman presented with APW and Eisenmenger syndrome. The patient underwent APW repair and bilateral lung transplantation. We transected the communication between the aorta and pulmonary artery and closed the aortic side directly with strips of felts. Nine months after the surgery, the patient complained of chest pain. Cardiac computed tomography revealed an ascending aortic pseudoaneurysm at the anastomotic site. Emergent graft replacement of the ascending aorta was performed and the postoperative course was uneventful.

**Conclusions:**

We have presented a case of a pseudoaneurysm at the anastomotic site after APW repair and bilateral lung transplantation. The choice of surgical technique should be based on the patient’s background requiring lung transplantation, and in these cases close postoperative follow-up is required.

## Background

Aortopulmonary window (APW) is a rare congenital cardiac anomaly characterized by communication between the main pulmonary artery and ascending aorta.

There are various surgical techniques, and the short- and long-term results are excellent if the surgical repair is performed early in life. To our knowledge, there are no reports of pseudoanuerysm after APW repair.

Herein, we present a case of a 30-year-old woman with an ascending aortic pseudoanuerysm, found at the anastomotic site nine months after APW repair and bilateral lung transplantation.

## Case presentation

A 30-year-old woman was diagnosed with an APW and pulmonary hypertension at five years of age. She was registered for lung transplantation at the age of 23 years and underwent bilateral lung transplantation and APW repair at the age of 29 years. She was discharged from the hospital three months after the surgery.

Due to severe pulmonary hypertension, the right femoral artery and vein were secured to establish an emergent cardiopulmonary bypass (CPB) before the operation. Since the circulation was stable, the chest was opened without using ECMO. The wall of the ascending aorta was thin and unsuitable for arterial cannulation, so CPB was established through the right femoral arterial cannulation and bicaval venous cannulation.

The ascending aorta was cross-clamped distal to the APW, and cardiac arrest was achieved with antegrade cardioplegia from the ascending aorta.

**Fig. 1:**
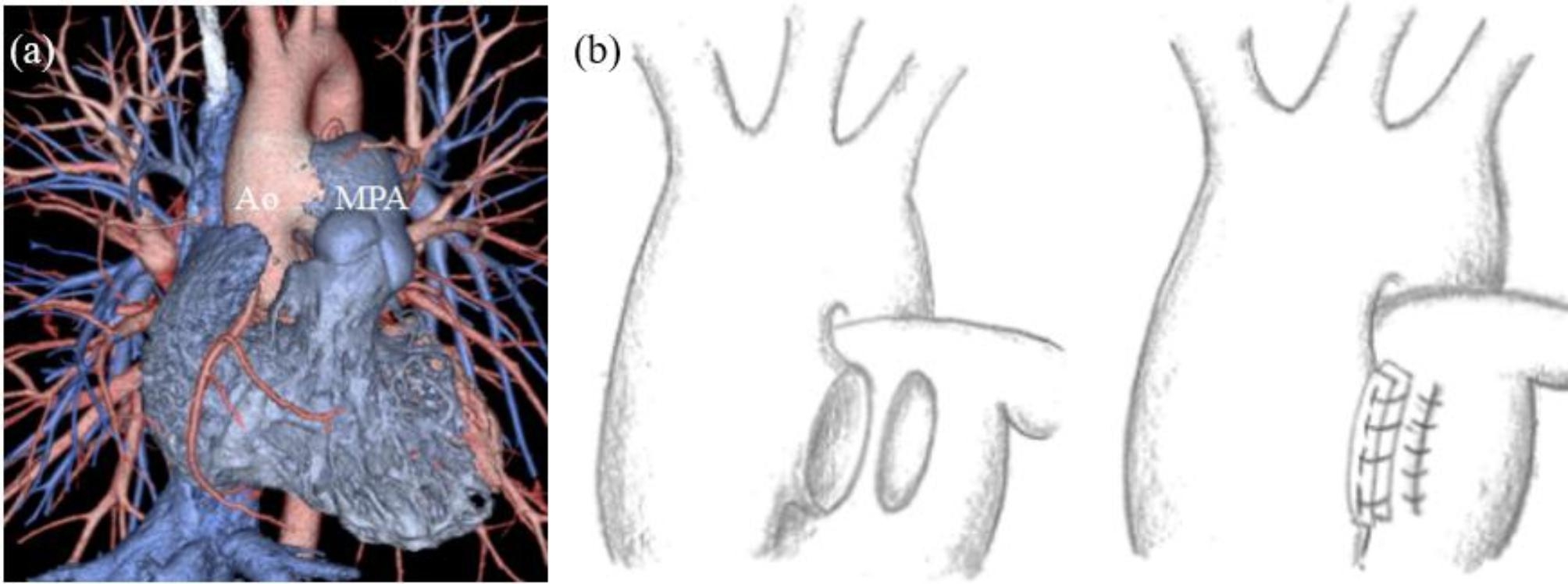
(**a**) Preoperative computed tomography. Preoperative computed tomography image showed aortopulmonary window. (**b**) Intraoperative findings. The aortopulmonary artery was dissected between the aorta and pulmonary artery. The dissection on the aortic side was sandwiched between two felts and directly closed with 4-0 Prolene sutures. In contrast, dissection on the pulmonary artery side was closed directly with 5-0 Prolene sutures Ao: aorta, MPA: main pulmonary artery

While repairing the APW, we dissected the traffic between the aorta and main pulmonary artery and directly closed the aortic side with the pulmonary artery wall and strips of felts (Fig. 1).

After the APW repair, the cross-clamp was released, and bilateral lung transplantation was performed.

Nine months after the surgery, she visited the previous physician with a complaint of chest pain and was referred to our hospital for further management of the pseudoaneurysm of the ascending aorta.

Blood tests revealed an elevated inflammatory response (white cell count, 10,930; C-reactive protein level, 4.9). Chest radiography, electrocardiogram, and echocardiography did not reveal any abnormalities.

**Fig. 2:**
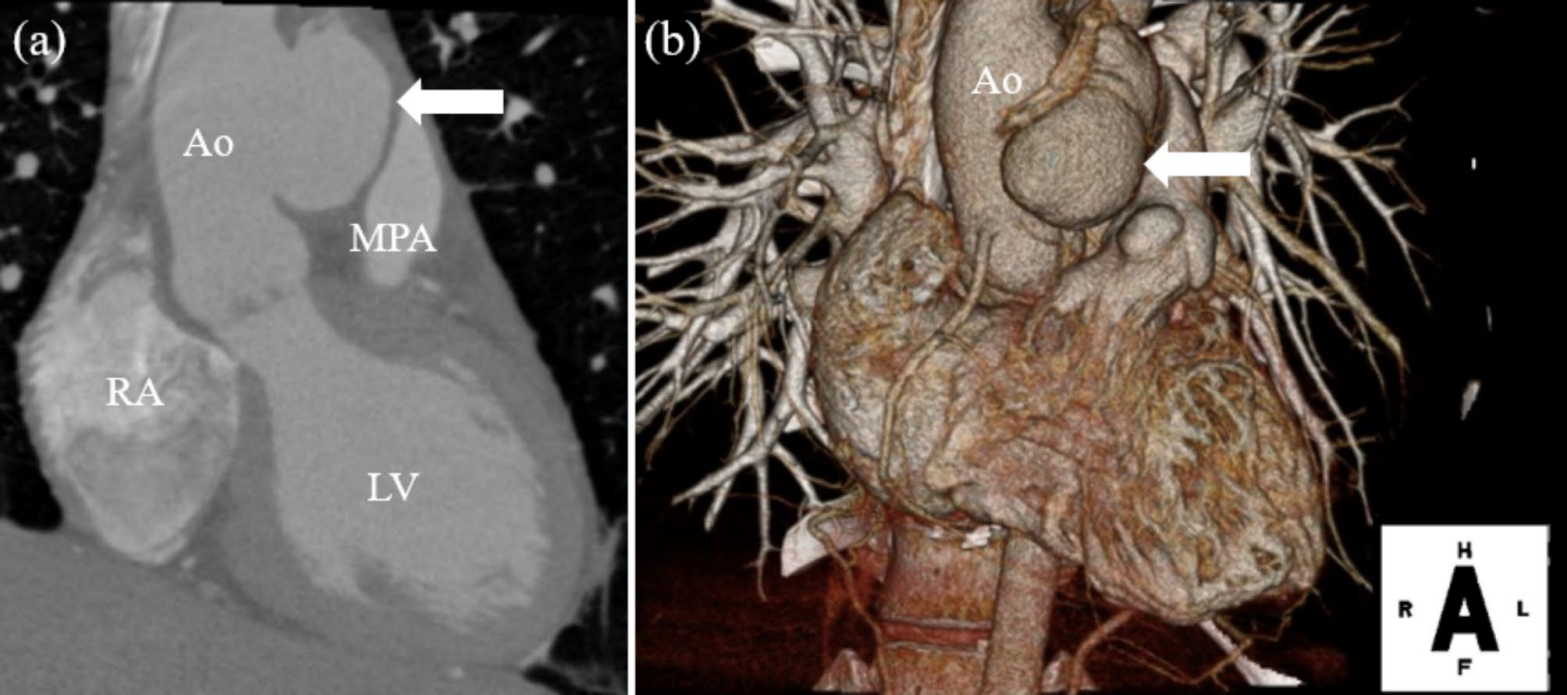
(**a**, **b**) Preoperative computed tomography images. CT showed a pseudoaneurysm of the ascending aorta (white arrow) Ao: aorta, MPA: main pulmonary artery, RA: right atrium, LV: left ventricle

Cardiac computed tomography (CT) showed an ascending aortic pseudoaneurysm in the repaired APW (Fig. 2).

We performed monthly or bimonthly CT follow-ups to examine the bilateral lungs following APW repair and bilateral lung transplantation before.

The aorta appeared normal until eight months after the surgery; however, a CT scan revealed a giant ascending aortic pseudoaneurysm.

Since the mass was rapidly increasing in diameter, and the risk of rupture was high, we performed an emergent graft replacement of the ascending aorta on the same day when she was transferred to our hospital.

Due to the risk of rupture of the pseudoaneurysm, CPB was established through the right femoral arterial cannulation and femoral venous cannulation before the redo sternotomy. There was severe adhesion in the mediastinum, and the pseudoaneurysm ruptured during dissection.

Under circulatory arrest at 20℃, the ascending aorta was cross-clamped at the root of the brachiocephalic artery, and then circulation was resumed.

**Fig. 3:**
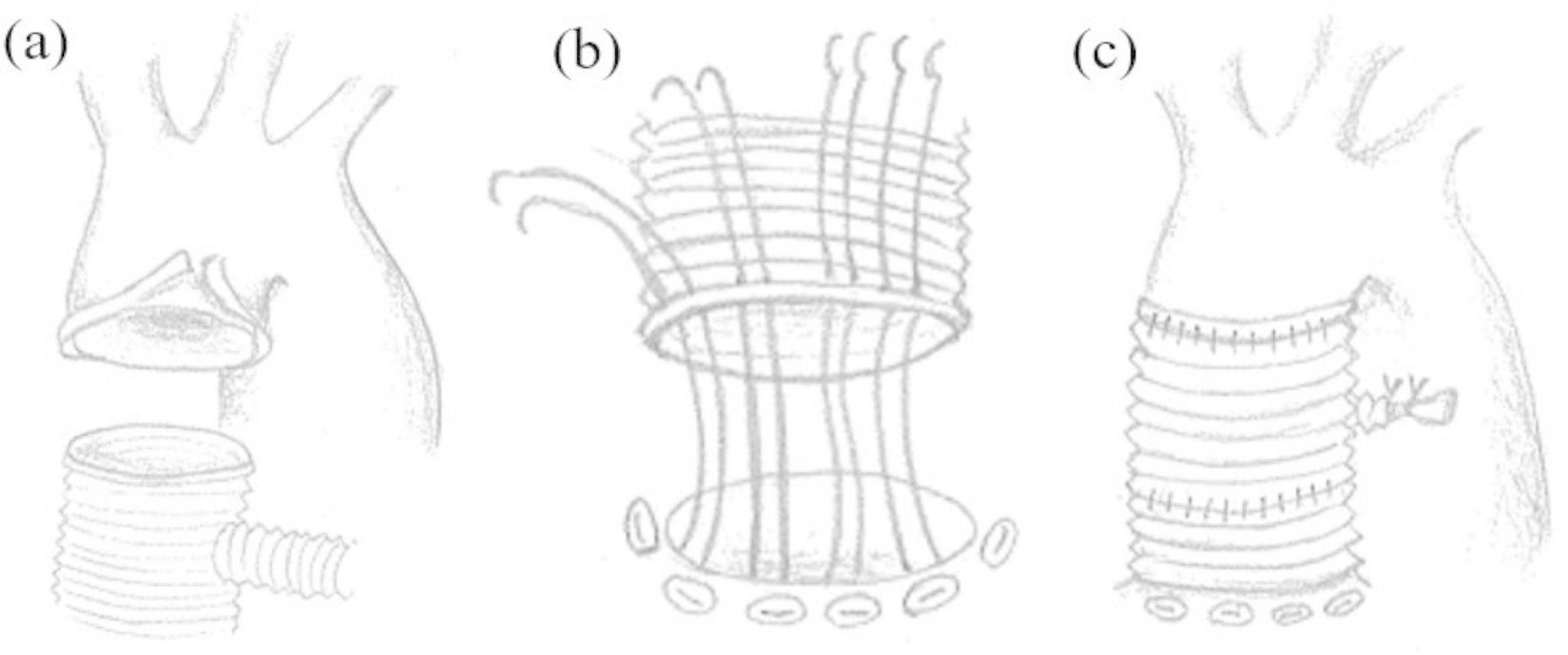
Operative schema (**a**) Peripheral anastomosis. Peripheral anastomosis between native aorta and a 20 mm-woven graft under circulatory arrest. (**b**) Proximal anastomosis. Proximal anastomosis was performed using ten interrupted everting mattress pledgeted sutures with felts and continuous sutures. (**c**) Anastomosis between proximal and distal grafts

The aneurysm wall was incised. There was no pus or infection around the aneurysm. Again, under the circulatory arrest, peripheral anastomosis was performed. The normal aorta was thin, so the anastomosis site was reinforced with a felt and anastomosed with a 20-mm woven graft (Fig. 3a). After retrograde cerebral perfusion, circulation was resumed. The proximal anastomosis was performed with a 20-mm woven graft using ten interrupted everting mattress pledgeted sutures with felts and continuous sutures (Fig. 3b).Finally, the proximal and peripheral grafts were continuously sutured (Fig. 3c).

The patient had a favorable postoperative course. She was extubated on the day of the surgery and discharged from the intensive care unit on postoperative day 5. Postoperative CT and echocardiography findings were unremarkable, and the patient was discharged on postoperative day 11.

Pathology of the resected ascending aortic aneurysm wall did not show any evidence of cystic medial necrosis.

Nonspecific chronic inflammation of lymphocytes was found in media and adventitia .

## Discussion and conclusions

APW is a congenital heart disease with traffic between the ascending aorta and pulmonary artery, accounting for 0.2% of all the congenital heart diseases [1]. APW leads to pulmonary hypertension and Eisenmenger syndrome without early surgery, and surgical repair is not possible [2]. In the present case, the patient was five years old at the time of APW diagnosis, leading to Eisenmenger syndrome; therefore, surgery was not indicated. At 19 years of age, the patient started treatment for pulmonary hypertension with pulmonary vasodilators. The patient was then registered for lung transplantation, and APW repair was planned at the time of lung transplantation.

Although a case of APW with ascending aortic aneurysm complications has been reported [3], to our knowledge, this is the first report of a pseudoaneurysm at the anastomotic site after APW repair.

In the present case, the aortopulmonary artery traffic area was dissected on the pulmonary artery side and closed directly between the aortic sides with strips of felts.

The reasons for choosing this procedure instead of patch repair and graft replacement of the ascending aorta are described below.

The short- and long-term postoperative results of a simple APW correction in childhood are excellent, and the occurrence of postoperative pseudoaneurysm has not been reported [2, 4–6].

Wound healing is delayed, and the patient is prone to infection due to the use of immunosuppressive drugs and steroids after bilateral lung transplantation. In addition, graft replacement of the ascending aorta is more invasive because it involves cardiac and circulatory arrest.

Patch closure was not the first choice because an artificial material was needed. In this case, the communication on the aortic side was situated away from the aortic valve commissure, so direct closure could be performed without causing aortic regurgitation.

For these reasons, we decided that a direct closure, a less invasive procedure without artificial blood vessel, was appropriate. However, this resulted in an anastomotic pseudoaneurysm at the direct closure site.

Common causes of anastomotic pseudoaneurysms include arterial wall degeneration, arterial fragility, suture failure, infection, and anastomotic technique [7].

There are three possible causes of the pseudoaneurysm in the present case. First, arterial fragility could contribute to the pseudoaneurysm.

The normal aortic wall was thin and could not withstand even the normal blood pressure to the anastomosis area. Second, use of steroids and immunosuppressive drugs after lung transplantation may have caused delayed wound healing at site of the direct closure on the aortic side. Third, a failure to match the intima of blood vessels to each other at the time of direct closure on the aortic side during APW repair. Intraoperative and pathological findings ruled out an infectious aneurysm.

In the present case, CT did not show pseudoaneurysm eight months after the procedure, and a pseudoaneurysm was first observed nine months after the procedure.

Since it was not a pseudoaneurysm in the acute postoperative period, it was more likely due to arterial fragility and delayed wound healing caused by postoperative steroids and immunosuppressive medications than due to the anastomotic technique.

In the present case, with delayed wound healing and fragility of the aortic wall, reconstruction with an artificial blood vessel could be more desirable than reconstruction with only fragile autologous tissue.

To conclude, we have presented a case of a pseudoaneurysm at the anastomotic site after APW repair and bilateral lung transplantation.

The choice of surgical technique should be based on the patient’s background requiring lung transplantation, and close postoperative follow-up is needed.

## Data Availability

Data sharing is not applicable to this article as no dataset were generated or analyzed during the current study.

## List of Abbreviations

APW: Aortopulmonary window
CPB: Cardiopulmonary bypass
CT: Computed tomography
